# Abundance, Diversity, and Distribution of Primates at Welel Mountain, Kellem Wollega Zone, Oromia Region, Ethiopia

**DOI:** 10.1155/2020/5691324

**Published:** 2020-05-11

**Authors:** Diriba Fufa, Dereje Yazezew, Gezahegn Degefe, Sibhatu Gebrehiwot

**Affiliations:** ^1^Department of Biology, College of Natural and Computational Sciences, Raya University, P.O. Box 92, Maichew, Ethiopia; ^2^Department of Biology, College of Natural and Computational Sciences, Debre Berhan University, P.O. Box 445, Debre Berhan, Ethiopia

## Abstract

Primates are the mammals of the order Primate that is characterized by advanced development of binocular vision and enlargement of the cerebral hemispheres. The aim of this study was to investigate the abundance, diversity, and distribution of primates on Welel Mountain. From August 2017 to February 2018, we collected data from different parts of Welel Mountain during wet and dry seasons of the year and analyzed them using SPSS version 20. We identified four primate species: *Chlorocebus aethiops, Cercopithecus mitis, Papio anubis,* and *Colobus guereza*. We conducted *t*-test analysis for abundance and distribution of primates in wet and dry season of the year, and the *P* value obtained was 0.20. The mean percentages of primates in forest, woodland, and shrubs were 43.16%, 32.26%, and 24.58%, respectively. Shannon-Wiener diversity index (H′) value was higher in wet season than in dry season. The current study showed that the species are distributed more evenly in wet season than in dry season, and the number of young individuals is more than that of adults. This indicates that currently the status of primates population on Welel Mountain is good. Therefore, to keep the status of primates in the study area effective, wildlife management and conservation policy should be formulated.

## 1. Introduction

Primates are not evenly distributed across the globe as well as within the regions and vary greatly from time to time due to several factors. Thus, the study of the abundance, diversity, and spatial distribution of organisms and similarly an understanding of the basic quantitative natural history of primate species are critical to their conservation [1]. Primates arose from ancestors that lived in the trees of tropical forests; many primate characteristics represent adaptations to life in the challenging three-dimensional environment [2].

Africa is a continent of particular concern in terms of global primate conservation for many reasons. Firstly, it harbors a high primate diversity—at least 64 species are recognized: 15 prosimians, 46 monkeys, and 3 apes [3], representing approximately 30% of extant primate species. Among fifteen countries worldwide scoring highest for primate species richness, nine are in Africa, comprising Cameroon, Democratic Republic of Congo (DRC), Nigeria, Peoples Republic of Congo, Equatorial Guinea, and Central African Republic [4]. Secondly, historically African forests have been highly dynamic, experiencing several cycles of expansion, and in many regions forests have persisted in fragmented form [5]. Large‐scale, historical processes (e.g., speciation, extinction, and dispersal) have been important in shaping the current patterns of primate distribution on the continent [6].

Primate habitats span a range of altitudes. For example, the *Rhinopithecus bieti* has been found living in the Hengduan Mountains at altitudes of 4,700 m [7], the *Gorilla beringei beringei* can be found at 4,200 m crossing the Virunga Mountains, and the *Theropithecus gelada* has been found at elevations of up to 5,000 m in the Ethiopian Highlands. The diversification of species is largely influenced by the conditions at the origin, and during the subsequent history of the clade [8], the conditions currently associated with higher levels of species richness can hint at the main environmental axis determining the distribution and diversification of that clade. In addition, contemporary conditions and events such as anthropogenic changes in habitat and climate continue to influence species diversity by removing species from some areas and adding them to others [9].

Accounting for 25% to 40% of the fruit-eating animals (by weight) within tropical rain forests, primates play an important ecological role by dispersing seeds of many tree species [10]. Africa is a continent of particular concern in terms of global primate conservation, for a variety of reasons [11]. Among fifteen countries worldwide scoring highest for primate species richness, nine are in Africa [12].

Globally, primate populations are being dramatically impacted by activities such as logging, deforestation, hunting, and other such factors. As a result, wild populations of most nonhuman primates (NHPs) are decreasing all over the world and many thousands of primates are killed every year for different purposes [13]. The aim of this study was determining diversity, abundance, and distribution of primates on Welel Mountain and formulating effective and realistic management policy to control illegal activities.

## 2. Methods

### 2.1. Study Site

**Figure 1:**
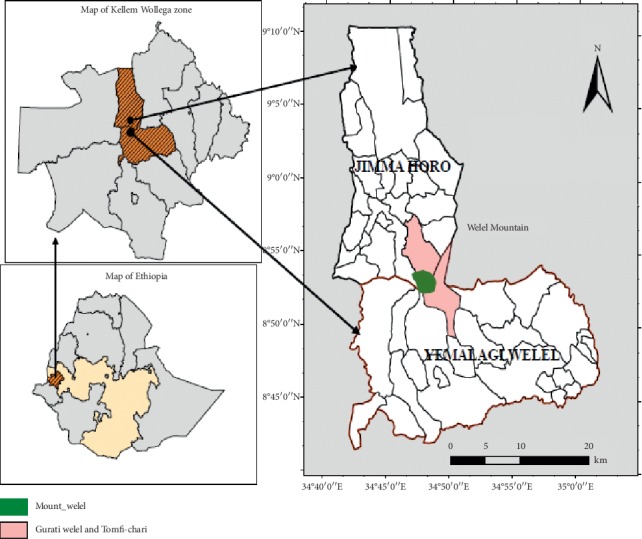
Map of the study area.

We conducted this study on Welel Mountain, Oromia Region, Ethiopia, situated 650 Km away from Addis Ababa to the west (see Figure 1). The area is located between 8° 5′–8° 8′ N latitude and 34° 5′–34°8′ E longitude. The altitude of the mountain is 3301 above sea level. The area receives over 3500 mm average annual rainfall [14]. The mean minimum and maximum temperature of the study area are 4°C and 20°C, respectively.

Welel Mountain forest contains diverse plant species. It harbors plant from lower level to large trees. Some of the plants are *Cordia africana*, *Ficus sur*, *Juniperus procera*, *Teclea nobilis*, *Grevillea robusta*, *Senecio gigas*, *Maesa lanceolata*, *Vepris dainellii*, *Croton macrostachys*, *Prunus africana*, *Arundinaria alpine*, *Vernonia myriantha*, *Dombeya torrid*, *Bersama abyssinica*, *Landolphia buchananii*, and many more plants. The following large mammal species are known to occur in the study area: *Tragelaphus scriptus*, *Crocuta crocuta*, *Colobus guereza*, *Papio anubis*, *Chlorocebus aethiops, Potamochoerus larvatus*, *Panthera pardus*, *Xerus* spp., *Sylvicapra grimmia*, *Orycteropus afer*, *Cercopithecus mitis*, *Leptailurus serval*, *Civettictis civetta*, *Hystrix cristata*, and others. In addition, the area contains many birds, amphibians, and reptiles.

### 2.2. Data Collection Methods

Firstly, the physical environment of the study area was observed, and preliminary surveys were made with local field assistants and the residents around the area who know the most common places where primates are found. Then, basic information about temperature, vegetation, and fauna of the study area was gathered. The data collection was conducted on Welel Mountain from August 2017 to February 2018 in wetland dry season. The wet season study included August 2017 to November 2017 and the dry season one included December 2017 to February 2018.

Data collection methods such as interviews, direct observations aided by naked eye and binocular (7 × 50 mm), and camera traps (Canon camera Eos 5d) were used to conduct the current study. In the study site, counting of the population of primates was carried out using direct observation while moving on foot throughout the whole study sites (three habitat types) which were divided into blocks before the counting of population. Primates in the area were counted by dividing the forest into blocks and each block was sampled by line transects [15].

Then the counting of primates seen from the transect lines continued and the primate seen in them was recorded [16]. Thirty transect lines were established, with 14 for riverine forest, 10 for woodland, 6 for shrub habitats depending on the area cover of each habitat. In riverine forest transect length of 2 km and width of 100 m, in woodland transect length of 2.5 km and width of 100 m, and in shrub transect length of 1.5 km and width of 100 m were used. Each transect in each habitat type was surveyed once every 2 months for six months (August 2017 to February 2018). Transects in each habitat were surveyed at the same time every morning and late afternoon at the time when most primates are active and have good visibility [14, 17].

These activities were done repeatedly during dry and wet season, and the primate's census was carried out five days per month for both wet and dry seasons. Then, primate's population was categorized into different age groups, namely, adult, subadult, and infant (juvenile); body size was used in age determination. Photographs of the primates were taken by means of digital camera, and the position at which they were counted was recorded. Observation was made by naked eye and by use of binocular. Then, the identified primates were counted and grouped as common (if probability of seeing is 100% in every time of field work), uncommon (if probability of seeing is > 50), and rare (if probability of seeing is < 50) [18]. Shannon-Wiener index (*H*′) which is given in the following formula was used to compare primates' diversity and similarity among habitats types and seasons, respectively [14, 19]:

(1) $$\begin{array}{c} H^{\prime }=-\sum \text{pi}\left(\ln\text{pi}\right), \end{array}$$

where *H*′ denotes the diversity indices and pi = number of individuals of species/total number of samples = number of species or species richness. Relative abundance is calculated by dividing the number of individuals of a species by the total number of individuals of all species.

Data were collected from different parts of Welel Mountain in wet and dry season of the year, and continuous field surveys were conducted during the field work period (covering several sites on Welel Mountain). The image files and pictures were taken with digital camera. Furthermore, audio files were recorded using a sound recorder; people were allowed to listen to sound recordings of primate species, and open-ended questions were additionally formulated so that the respondents were able to tell us about the species and anything that they thought was useful information.

### 2.3. Data Analysis

Statistical Package for Social Sciences (SPSS) version 20.0 (computer software for Windows, evaluation version program) and Microsoft Excel spreadsheets were used to analyze the data collected during the survey. One-way ANOVA was used to compare the relative abundance and distribution of primates in each habitat at 5% level of significance.

## 3. Results

### 3.1. Primate Species Identified in Different Habitats of Welel Mountain

**Figure 2:**
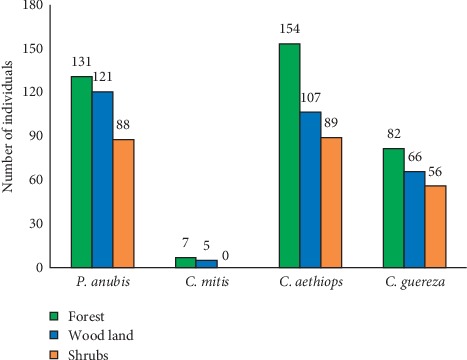
Number of primates at Welel Mountain in wet season.

**Figure 3:**
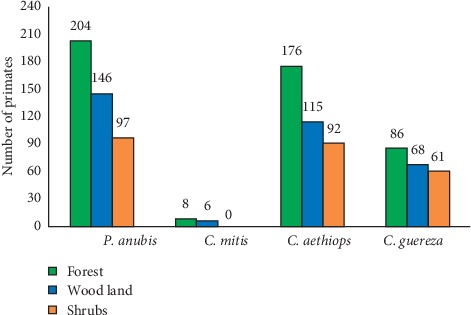
Number of primates at Welel Mountain in dry season.

A total of 4 species of primates, namely, *Chlorocebus aethiops*, *Cercopithecus mitis*, *Papio anubis*, and *Colobus guereza*, were observed at Welel Mountain through direct and indirect observations. Woodland habitat encompassed 66, 107, 5, and 121 individuals of *Colobus guereza*, *Chlorocebus aethiops*, *Cercopithecus mitis*, and *Papio anubis*, respectively, in wet season and 68, 115, 6, and 146 individuals of *Colobus guereza, Chlorocebus aethiops*, *Cercopithecus mitis*, and *Papio anubis*, respectively, in dry season. Riverine forest contained 86, 176, 8, and 204 individuals of *Colobus guereza, Chlorocebus aethiops*, *Cercopithecus mitis*, and *Papio anubis*, respectively, in dry season and 82, 154, 7, and 131 individuals of *Colobus guereza, Chlorocebus aethiops*, *Cercopithecus mitis*, and *Papio anubis*, respectively, in wet season whereas. Shrub land is inhabited by 61 *Colobus guereza*, 92 *Chlorocebus aethiops*, no *Cercopithecus mitis*, and 97 *Papio anubis* individuals during the dry season and 56 *Colobus guereza*, 89 *Chlorocebus aethiops*, no *Cercopithecus mitis*, and 88 *Papio anubis* individuals in wet season (see Figures 2 and 3).

### 3.2. Diversity Indices for Primates at Welel Mountain

The sum total of 1965 individual primates were counted and recorded in both wet and dry seasons. This means, on average 983 individuals were identified and recorded in the study area. From these, the total number of primates counted in wet season is less than that of dry season (see Table 1). The result from the interview revealed that there is migration of primates from the forest to the farmland during the wet season. Therefore, the number of primates counted in wet season is less than that of dry season. The woodland was represented by 634 individuals while riverine forest and shrubs were represented by 848 and 483 individuals, respectively. On average (wet and dry season), 43.16% of the total primates were counted in the forest, 32.26% were counted in woodland, and finally 24.58% were counted in shrubs. Moreover, relative abundance and diversity indices (*H*′) of each primate species in wet and dry season for the three habitat types were also calculated (see Table 1).

Shannon-Wiener (*H*′) value is 1.14 for wet season and 1.11 for dry season (see Table 1), which means that Shannon-Wiener diversity index (*H*′) value is higher in wet season than in dry season.

High value of Shannon-Wiener index (*H*′) is scored in forest habitat during dry season; this shows that forest harbors high number of individual primates. Moreover, high value of Shannon-Wiener index (*H*′) was observed in the forest during the wet season (see Table 2). This evidence suggests that primate species are more diversified and evenly distributed in the forest of Welel Mountain. Among the three habitat types, shrubs have the least diversified and distributed primate species.

## 4. Discussions

On average (in wet and dry season), we counted 43.16%, 32.26%, and 24.58% of the total primates in the forest, woodland, and shrubs habitats, respectively. Thus, forest habitat harbors the highest number of primate species. Distribution and habitat association of primates are determined in terms of their water and food requirements as well as suitability of foods. According to [20], there is food preference among different primate species and not all foods are suitable for them. Water and food or the combinations of both are the major factors determining the distribution of wildlife populations in their natural habitats [21].

However, the availability of these resources differs from season to season; as a result, the abundance and distribution of the primates differ from habitat to habitat since they prefer the area where all the life necessities are fulfilled. However, if the resources are not available enough, competition will be formed and migration will take place. Similarly, our current study revealed that there was migration of primates from forest to farmland during the wet season. This implies that some primates move to the farmland in search of food. This result is consistent with [22] who reported that the wild animals are increasing year by year which is due to competitions for resources between wild animals and human populations. This process can also cause conflict between primates and human population. Habitat destruction and fragmentation were the main cause of human-primate conflict in Indonesia [23].

The result of our study showed that the population of primates in the area varied from season to season. From all the four primate species that were identified in the study area, *Chlorocebus aethiops* and *Papio anubis* were relatively abundant during wet and dry season, respectively. This indicates that the ability of *Chlorocebus aethiops* and *Papio anubis* to exploit different varieties of food enables them to survive with large number of populations in the area. This goes in line with a study conducted by [24] who reported that one major reason for the wide spread of such primates is their feeding types. For example, dietary specialists and generalists may not be distributed equally. Similarly, a study conducted in Uganda demonstrated that the species are very flexible in terms of plant species and parts exploited for food [25, 26]. Hence, differences in diets among primate species may in part account for determining their distribution. Shannon-Wiener diversity index (*H*′) value is higher in wet season (1.14) than in dry season (1.11). However, numbers of individuals of primates counted in wet season are less than those of dry season. Here, Shannon-Wiener index value does not consider number of individuals only; instead it considers both species evenness and species richness.

Relatively more primate population was recorded during the dry season than the wet season, because the availability of food is more on the farmland than the forest (on the mountain) during the wet season, and the farmland across the forest becomes attractive and provides a plenty of food sources for these primates. Therefore, during the wet season, there is migration of primate populations from the forest, woodland, or shrubs to the farmland. Nevertheless, during the dry season, food becomes scarce in the farmland, and thus the primates might temporally migrate back from farmland to forest, woodland, or shrubs. This result is in line with a study conducted by [27] who reported that more primate population was recorded on farmland during the wet season than the dry season.

Our study results show that riverine forest has supported the highest number of primate species which is 374 individuals in wet season and 474 individuals in dry season, followed by woodland which comprised 299 individuals in wet season and 335 in dry season, and shrub land habitat harbored 233 individuals in wet season and 250 in dry season. From the result of the current study, the calculated *P* value for distribution of primates with regard to habitats is 0.58. This value is greater than *α* (0.05), and it shows that, although there is a difference in number of individuals among the three habitats, there is no significant difference between the three habitats based on the abundance of primate species. Similarly, the calculated *P* value obtained from the comparison of primates in wet and dry season is 0.2, which is greater than 0.05. This shows that statistically there is no significant difference between the mean numbers of primates in wet and dry season.

In addition, a high value of Shannon-Wiener index (*H*′) was observed in woodland in case of dry season and in forest in case of wet season. The possible reason for this distribution and diversity might be the availability of food and water with regard to seasonal variation. The olive baboons (*Papio anubis*) were relatively the most abundant species in the present study area.

This may be due to the ability of *Papio anubis* to exploit different varieties of food more than other primates in this study. This goes in line with [24] reporting that one major reason for olive baboon's widespread success is that it is omnivorous. The olive baboon searches as wide area as it can, and it eats virtually everything it finds including small animals.

Similarly, the olive baboon in shrubs goes about finding food differently from that in the surrounding forest. As such, it is able to find nutrition in almost any environment, and it is able to adapt with different foraging tactics. The baboon forages on all levels of an environment, above and beneath the ground and in the canopy of forests, but most animals only look for food at one level; an arboreal species such as a lemur does not look for food on the ground [28]. Olive baboon appeared to be more concentrated in the riverine forest; besides, it was also recorded in woodland and, in least number, in shrub land.

The second most dominant primate species in the study area was *Chlorocebus aethiops*, which is widely distributed and often a common species in different parts of Ethiopia, occupying a wide variety of habitats ranging from riverine, tropical deciduous or montane forest to comparatively open woodland. In many areas, this monkey frequents human settlements and feeds extensively on cultivated plants. The species has been recorded near sea level and extends to an altitude of at least 3000 m [29]. In line with [29] in this study the species was observed mostly in riverine forest habitat. This association of *Chlorocebus aethiops* (grivet monkey) with riverine forest might be due to the availability of fruit tree species.

In contrast to other primates, colobus monkey was more frequently seen on the top of trees during the day and night, and usually it cannot feed on agricultural crops. This adaptation might prevent the species from human and predator's damage. In some areas, the species survives in relatively small patches of remnant forest and it is, in general, tolerant of the presence of man [29]. This might correlate with the habit that colobus monkey was more frequently seen on the top of tree and it cannot damage and feed on agricultural crops.

Lastly, blue monkey (*Cercopithecus mitis boutourlinii*) was relatively observed in more number in forest than other habitats in the study area. According to [30], the *boutourlinii* blue monkey groups were inhabiting relatively undisturbed and tree-dominated forest at Jibat, but venturing out into nearby bush land only occasionally to feed on some fruits. This subspecies was recently listed as vulnerable by IUCN because of extensive and uncontrolled destruction of the forests it occupies for both timber and agricultural production [31]. This may be because their habitat preferences are more than those of other primates. In the case of the present study area, more count of blue monkey was done in forest than other habitat types. According to [32], blue monkeys are among Africa's arboreal primate species and inhabit a variety of forest types (tropical moist forests). Moreover, in the current study more numbers of adult and subadult primates were counted. This indicates that the primate population of Welel Mountain are currently on good status and their number will increase in the future.

## 5. Conclusion

We identified four primate species of Welel Mountain, namely, *Chlorocebus aethiops*, *Cercopithecus mitis*, *Papio anubis*, and *Colobus guereza*. The four primate species identified on Welel Mountain have different habitat preferences. The findings obtained from this study indicated that the number of individual primates counted in wet season is less than that of dry season. These differences in distribution of primate's population may be due to the availability of water and food in different seasons. Therefore, water and food availability or the combinations of both are the major factors determining the abundance and distribution of primates. Since the availability of these resources differs from season to season as well as from habitat to habitat, the abundance and diversity of the primates vary. As a result, during the wet season there is migration of primate populations from the forest, woodland, or shrubs to the farmland in search of food. Based on the age category, more number of subadult primates was counted. From this, we can predict that their number will increase in the future. The current study showed that, from the all primate species identified in the study area, *Chlorocebus aethiops* and *Papio anubis* were relatively more abundant during wet and dry season, respectively. This is due to the ability of *Chlorocebus aethiops* and *Papio anubis* to exploit different varieties of food, which enables them to survive, and this may in part account for determining their distribution. *Cercopithecus mitis* were observed relatively in least number. This may be because their habitat preferences are more than those of other primates of this area.

Accordingly, to keep the good status of primates in the study area, the policymakers and wildlife managers should

- formulate effective wildlife management and conservation policy,
- implement appropriate measures of controlling regular clearing of the plant species in the study area,
- carry out primate population censuses in the study area at specific time to determine their population trends,
- conduct ecological research about the feeding behavior and diurnal activity on the species since it is essential to know the ecological relationship of the species with their respective environment and habitats.

## Figures and Tables

**Table 1:** Species richness, relative abundance, and diversity indices (*H*′) of each primate species in wet and dry season.

Types of primate species at Welel Mountain	In wet season			In dry season		
	No. of individuals	Relative abundance (pi)	*H*′	No. of individuals	Relative abundance (pi)	*H*′
*C. guereza*	204	0.23	0.34	215	0.20	0.32
*C. aethiops*	350	0.39	0.37	383	0.36	0.37
*P. anubis*	340	0.38	0.37	447	0.42	0.36
*C. mitis*	12	0.01	0.06	14	0.01	0.06
			1.14			1.11

**Table 2:** Species richness, diversity indices (*H*′), and relative abundance of total primates in each habitat type during the wet and dry seasons.

Wet season					Dry season				
Habitat	Primate species	No. of individuals	Relative abundance	Shannon diversity index (*H*′)	Habitat	Primate species	No. of individuals	Relative abundance	Shannon diversity index (*H*′)
Forest	A	82	0.22		Forest	A	86	0.18	
	B	154	0.41	1.14		B	176	0.37	1.11
	C	7	0.02			C	8	0.02	
	D	131	0.35			D	204	0.43	
	Total	374				Total	474		

Woodland	A	46	0.43		Woodland	A	68	0.20	
	B	107	0.38			B	115	0.34	
	C	5	0.02	1.1		C	6	0.02	1.12
	D	121	0.43			D	146	0.44	
	Total	279				Total	335		

Shrubs	A	56	0.24		Shrubs	A	61	0.24	
	B	89	0.38	1.08		B	92	0.37	1.08
	C	0	0.00			C	0	0.00	
	D	88	0.38			D	97	0.39	
	Total	233				Total	250		

A: *Colobus guereza*, B: Chlorocebus aethiops, C: *Cercopithecus mitis*, D: *Papio anubis*.

## Data Availability

The data used to support the findings of this study are available from the corresponding author upon request.

## References

[1] Fashing P. J. (2007). Behavior, ecology, and conservation of colobine monkeys: an introduction. *International Journal of Primatology*.

[2] Groves C., Wilson D., Reeder D. (2005). Order primates. *Mammal Species of the World: A Taxonomic and Geographic Reference: 111–184*.

[3] IUCN (1996). *African Primates: Status Survey and Conservation Action Plan*.

[4] Colin A., Chapman M. J., Harriet A. (2006). What hope for African primatediversity?. *African Journal of Ecology*.

[5] Hamilton A. C., Taylor D. (1991). History of climate and forests in tropical Africa during the last 8 million years. *Tropical Forests and Climate*.

[6] Lawes M. J., Eeley H. A. C. (2000). Are local patterns of anthropoid primate diversity related to patterns of diversity at a larger scale?. *Journal of Biogeography*.

[7] Long Y., Kirkpatrick C. R., Zhongtai T., Xiaolin L. (1994). Report on the distribution, population, and ecology of the Yunnan snub-nosed monkey (*Rhinopithecus bieti*). *Primates*.

[8] Romdal T. S., Araújo M. B., Rahbek C. (2013). Life on a tropical planet: niche conservatism and the global diversity gradient. *Global Ecology and Biogeography*.

[9] Vitousek P. M., Mooney H. A., Lubchenco J., Melillo J. M. (1997). Human domination of Earth’s ecosystems. *Science*.

[10] Chapman C., Russo S., Campbell C. J., Fuentes A., MacKinnon K. (2007). Primate seed dispersal. *Primates in Perspective*.

[11] Grubb P., Butynski T. M., Oates J. F. (2003). Assessment of the diversity of African primates. *International Journal of Primatology*.

[12] Cowlishaw G., Dunbar R. (2000). *Primate Conservation Biology*.

[13] Mittermeier R. A., Oates J. F., Eudey A. E., Thornback J., Mitchell G., Erwin J. (1986). Primate conservation: 3–72. *Comparative Primate Biology*.

[14] Gonfa R., Tsegaye G., Tadesse H. (2015). Diversity abundance and habitat association of medium and large sized mammals of Dati Welel National park, western Ethiopia. *International Journal of Biodiversity and Conservation*.

[15] Anderson D. R., Laake J. L., Crain B. R., Burnham K. P. (1979). Guidelines for line transect sampling of biological populations. *The Journal of Wildlife Management*.

[16] Plumper A. J., Reynolds V. (1994). The impact of selective logging on the primate populations in the Budongo forest reserve, Uganda. *Journal of Applied Ecology*.

[17] Peres C. (1999). General guidelines for standardizing line-transect surveys of tropical forest primates. *Neotropical Primates*.

[18] Hillman J. C. (1993). *Ethiopia: Compendium of Wildlife Conservation Information*.

[19] Krebs C. J. (1978). *Ecology: The Experimental Analysis of Abundance and Distribution*.

[20] Fashing P. J., Nguyen N., Luteshi P., Opondo W., Cash J. F., Cords M. (2012). Evaluating the suitability of planted forests for African Forest Monkeys: a case study from Kakamega forest, Kenya. *American Journal of Primatology*.

[21] Balakrishinan M., Easa P. (1986). Habitat preference of large mammals in the parambikulam wildlife sanctuary, Kerala, India. *Journal of Biological Conservation*.

[22] Hill C. M. (2000). Conflict of interest between people and baboons; crop raiding in Uganda. *International Journal of Primatology*.

[23] Priston N. (2001). Assessment of crop damage by Macacao chreata brunnescens in south east Sulawesi a farmer’s perspective.

[24] Cawthon L. K. (2006). Primate factsheets: olive baboon (*Papio anubis*) taxonomy, morphology & ecology. http://pin.primate.wisc.edu/factsheets/entry/olive_baboon.

[25] Chapman C. A., Chapman L. J., Gillespie T. R. (2002). Scale issues in the study of primate foraging: red Colobus of Kibale national park. *American Journal of Physical Anthropology*.

[26] Isabirye-Basuta G. M., Lwanga J. S. (2008). Primate populations and their interactions with changing habitats. *International Journal of Primatology*.

[27] Yihune M., Bekele A., Tefera Z. (2009). Human-gelada baboon conflict in and around the Simien mountains national park, Ethiopia. *African Journal of Ecology*.

[28] Whiten A., Byrne R. W., Barton R. A., Waterman P. G., Henzi S. (1991). *Dietary and Foraging Strategies of Baboons*.

[29] Yalden D. W., Largen M. J., Kock D. (1976). Catalogue of the mammals of Ethiopia. *Monitore Zoologico Italiano. Supplemento*.

[30] Dereje T., Peter J. F., Afework B., Addisu M., Anagaw A. (2013). Ecological flexibility in Boutourlini’s blue monkeys (*Cercopithecus mitis boutourlinii*) in Jibat forest, Ethiopia: a comparison of habitat use, ranging behavior, and diet intact and fragmented forest. *International Journal of Primatology*.

[31] Butynski T., Gippoliti S. (2017). *Cercopithecus mitis boutourlinii ssp.* IUCN red list of threatened species. http://www.iucnredlist.org.

[32] Butynski T. M. (1990). Comparative ecology of blue monkeys (*Cercopithecus mitis*) in high- and low-density subpopulations. *Ecological Monographs*.

